# Functional Outcome of Bipolar Prosthesis versus Total Hip Replacement in the Treatment of Femoral Neck Fracture in Elderly Patients

**DOI:** 10.5704/MOJ.1703.002

**Published:** 2017-03

**Authors:** R Shukla, M Singh, RK Jain, P Mahajan, R Kumar

**Affiliations:** Department of Orthopaedics, Sri Aurobindo Institute Medical Science, Indore, India

**Keywords:** bipolar prosthesis, femur neck fracture, THR, Harris Hip Score

## Abstract

**Introduction:**

The present study was performed to compare cemented total hip replacement (THR) with cemented bipolar prosthesis in the treatment of displaced fracture neck of femur in elderly patients.

**Materials and Methods:**

This prospective study included 47 patients of greater than 60 years of age and having fracture of neck of femur, out of which 25 patients were managed by cemented bipolar prosthesis and remaining 22 were managed by cemented THR between June 2011 and June 2013. These patients were followed up post-operatively for two years, at 6, 12 and 24 months, for functional analysis using Modified Harris Hip Score.

**Results:**

Modified Harris Hip Score was significantly higher in the THR group as compared to the bipolar prosthesis group at 6, 12 and 24 months post-operatively. Pain was almost similar in both the groups at all follow-up periods. Gait and range of motion was significantly higher in THR group as compared to bipolar prosthesis group at all-time point intervals.

**Conclusion:**

Cemented THR is a better option as compared to cemented bipolar prosthesis based on our short term functional outcome for the management of fracture of neck of femur in elderly patients.

## Introduction

Fracture of the neck of femur is one of the most common injuries in the elderly population leading to morbidity and mortality among them^1^. For the healthcare system and to society in general, femur neck fracture poses an epidemic problem. In the elderly with osteoporotic bones, a trivial fall is the cause of hip fractures in about 90% of cases^2^.

A typical patient with fracture neck of femur is characterized by old age, severe osteoporosis and significant co-morbid disease^3^-^5^. Fracture neck of femur has always been a great challenge to the orthopaedic surgeon and still remains the unsolved mystery as far as the treatment and its results are concerned.

The primary aim of treatment should be to perform a surgery that provides the patient with the greatest opportunity for early ambulation. This requirement is fulfilled to a great extent by the use of a primary prosthetic replacement either bipolar prosthesis or total hip replacement (THR). Therefore, the aim of the present study was to assess patients with displaced fracture neck of femur treated with either THR or bipolar prosthesis using modified Harris Hip Score and compare their results.

## Materials and Methods

This prospective study was carried out in the Department of Orthopaedics of a tertiary care center. Prior to the study institutional ethical clearance was obtained.

Patients with displaced fractures neck of femur who came to the emergency department were admitted in this hospital and treated surgically. We collected records of the patients by taking the patients’ history and examining them. All the patients were carrying out activities of daily living on their own prior to trauma. Patients with open fractures, suspected pathological fracture, and any other associated fracture and head injury were excluded from the study. Those patients who were unable to afford the surgical charges were also not included in the study. To identify a 5-point difference in the Harris Hip Score with 90% power of sample and based on previous study means^5^ sample size of minimum 13 patients in each group was required.

**Fig. 1:**
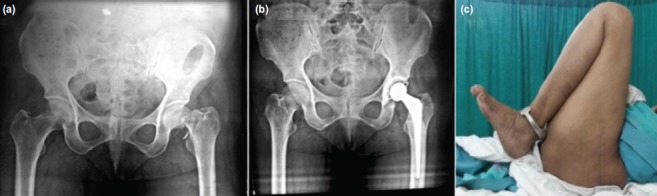
(a) Pre and (b) Post op radiographic images of patient treated with bipolar prosthesis for femur neck fracture. (c) Clinical picture of patient after one year of treatment.

**Fig. 2:**
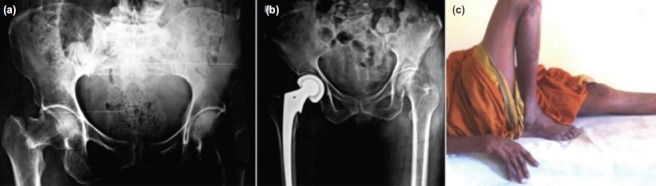
(a) Pre and (b) Post op radiographic images of patient treated with total hip replacement for femur neck fracture. (c) Clinical picture of patient after 1 year of treatment.

Before surgery patients were divided into the following two groups using random number table generated by GraphPad online software. Group 1 was treated with cemented bipolar prosthesis and Group 2 with cemented THR. A total of 47 patients were included in the study, out of which 25 were managed with cemented bipolar prosthesis (Fig. 1) and remaining 22 with cemented THR (Fig. 2). Detailed history was taken with particular emphasis on the mode of injury and associated medical illness. In-depth clinical assessment was carried out in each case. In all patients, preoperatively skin traction was applied to the affected lower limb for 2-3 days, with the aim of relieving pain, preventing shortening and to reduce unnecessary movements of the injured limb. Oral or parenteral analgesics were given to relieve pain. Antero-posterior radiographs of the affected hip joint of pelvis with both hips were taken for all the patients, keeping the fractured limb in 15 degrees of internal rotation to bring the neck parallel to the radiograph film. Patients as well as the next-of-kin were explained about the surgery and risk factors and expenses, and written informed consent for the surgery was obtained from all patients. Both the surgical procedures were done using standard Moore's (southern) posterior approach. In most of the patients; surgery was performed within 2-3 days after passing fitness for anaesthesia.

Post-surgical rehabilitation was similar for both groups and consisted of a joint-care programme rehabilitation protocol. Full weight-bearing and active exercises were commenced within first three days after surgery as tolerated. Patients were discharged after 5-7 days as per standard protocol and rehabilitated during the study period. Exercises for active muscle strengthening were advised and range of motion was tested. Some life style modifications were advised to all the patients. For functional assessment, Modified Harris Hip Score was assessed at 6, and 12 and 24 months post operatively.

Data were entered in Microsoft Excel and analyzed on MedCalc Statistical Software (Trial Version). Mann Whitney U test was used for comparing the age, duration of surgery, blood loss and Harris Hip Score parameters in the two groups. Chi Square test was used to see the difference in gender between the two groups. A p value <0.05 was considered significant.

## Results

There were 47 (30 female; 17 male) patients in our study, out of which 25 patients (16 female; 9 male) underwent bipolar prosthesis treatment and 22 (14 female; 8 male) underwent THR treatment. All the patients in both the groups were above the age of 60 years. The mean age of patient was 68.3 years in the bipolar group and 65.3 years in the THR group (Table I).

**Table I:** Demographic profile of the two groups

	Bipolar prosthesis (25)	THR (22)	p Value
Age	68.3 ± 6.5	65.36 ± 6.3	0.1235
Sex (Male)	9 (36)	8 (36.36)	0.9793
Average waiting days (Days)	3.9±1.2	4.5±1.1	0.0822
Hypertension	9 (36)	6 (27)	0.550
Diabetes mellitus	4 (16)	4 (18)	1.000
Anemia	6 (24)	6 (27)	1.000
Chronic obstructive pulmonary disease	2 (8)	4 (18)	0.398

The common problems in our series were gross anemia, hypertension, diabetes mellitus, chronic bronchitis and bronchial asthma. Twenty-three patients (48.9%) in the study had one or more of the problems.

Mean duration of surgery in the THR group (110.00 min) was significantly higher than the mean duration of surgery in the bipolar prosthesis group (82.12 min) (p< 0.0001). The mean blood loss in the THR group (468.18 ml) was significantly higher than the mean blood loss in the bipolar prosthesis group (320.40) (p = 0.015). Thus, the bipolar prosthesis treatment was found better than THR in relation to duration of surgery and total blood loss during surgery (Table II).

**Table II:** Surgical profile of the two groups

	Bipolar prosthesis	THR	p Value
Duration of surgery	82.12 ± 22.77	110.00 ± 18.71	<0.0001
Blood loss	320.40 ± 126.70	468.18 ± 239.42	0.015

At six months, the Harris Hip Score was measured in both the groups. The total score in the bipolar prosthesis group was 74.68 in comparison to 80.68 in the THR group. The difference was significant (p value < 0.0001). At six months, the Harris Hip Score in THR group was better than in the bipolar prosthesis group. There was no significant difference in the mean pain score of bipolar prosthesis group or the THR group (p value = 0.083). Gait score, activity score and range of motion was significantly higher in THR than bipolar prosthesis group (Table III).

**Table III:** Mean Harris Hip Score for all patients in both the groups at six months

	Bipolar prosthesis	THR	p Value
Total Score	74.68±5.46	80.68±1.86	<0.0001
Pain	38.80±3.32	40.00±0.00	0.083
Function			
a. Gait	20.56±2.24	23.41±1.05	<0.0001
b. Activity	7.40±1.00	8.41±1.44	0.009
Range of motion	7.92±0.28	8.86±0.35	<0.0001

At 12 months, the Harris Hip Score was measured in both the groups. The total score in the bipolar prosthesis group was 78.24 compared to 84.73 in the THR group. The difference was significant (p value < 0.0001). At 12 months Harris Hip Score in THR group was better than the bipolar prosthesis group. The mean pain score was 39.20 in the bipolar group and 40.00 in the THR group (p value =0.162). The mean gait score was 22.12 in the bipolar prosthesis group in comparison to 26.55 in the THR group and was highly significant (p value< 0.0001). The mean activity score was 8.84 in bipolar prosthesis group in comparison to 9.45 in THR group and was not statistically significant (p value = 0.178) (Table IV).

**Table IV:** Mean Harris Hip Score for all patients in both the groups at 12 months

	Bipolar prosthesis	THR	p Value
Total Score	78.24±4.28	84.73±1.91	<0.0001
Pain	39.20±2.77	40.00±0.00	0.162
Function			
a. Gait	22.12±1.51	26.55±2.97	<0.0001
b. Activity	8.84±1.43	9.45±1.60	0.178
Range of motion	8.24±0.44	8.77±0.43	<0.0001

At 24 months, the Harris Hip Score was assessed in both the groups. The total score in the bipolar prosthesis group was 81.40 in comparison to 89.32 in the THR group. At 24 months, the Harris Hip Score in THR group was better than the bipolar prosthesis group (p value <0.0001). There was no significant difference in the mean pain score in the bipolar prosthesis group and THR group (p value = 1.000). Gait score, activity score and range of motion was significantly higher in THR than bipolar group at 24 month of follow up (Table V).

**Table V:** Mean Harris Hip Score for all patients in both the groups at 24 months

	Bipolar prosthesis	THR	p Value
Total Score	81.40±2.77	89.32±0.57	<0.0001
Pain	40.00±0.00	40.00±0.00	1.000
Function			
a. Gait	23.36±1.32	29.32±1.29	<0.0001
b. Activity	9.76±1.67	10.91±1.80	0.029
Range of motion	8.44±0.51	9.00±0.00	<0.0001

## Discussion

Femoral neck fractures are common injuries among elderly people^1^. The common treatment for a displaced femoral neck fracture in the elderly is replacement of the femoral head. The arthroplasty can be either bipolar prosthesis or THR. The question of whether a bipolar prosthesis or THR has been a topic of controversy and ongoing debate. In this context, we undertook the present study to evaluate the results of cemented bipolar prosthesis or cemented THR in fracture neck of femur with two years follow-up. The results were analyzed at 6, 12 and 24 months and observations were made.

The elderly females are more prone to fracture neck of femu^2^-^5^. Male preponderance is reported in few series^6^. In our study female preponderance was 64% in the bipolar group and 63.64% in the THR group.

The common coexisting illnesses in our series was gross anemia, hypertension, diabetes mellitus, chronic bronchitis and bronchial asthma. Ischaemic heart diseases were common in Western series, but was not so in our series. The patients with nervous system disorder and mental problems were not observed in our study whereas they were common in Western series.

In our study, we had one (4%) superficial infection in the bipolar group and one (4.5%) superficial infection in the THR group. Blomfeldt *et al*^7^ reported two cases of superficial infection in both the groups and one case of deep infection which required wound debridement. No case of deep infection was noted in our study. Superficial infection was seen in the patients who were diabetic and anaemic. They developed signs of infection in the first post-operative week. They were treated with appropriate antibiotics and dressings. All these infections were found when the patients were still in the hospital and this resulted in prolongation of their hospital stay. In our study there was no incidence of peri-prosthetic fracture, while one patient of THR group developed peri-prosthetic fracture in similar study by Blomfeldt *et al*^7^

There were no dislocations in any patient in our study. This matches with the results of similar study by Blomfeldt *et al*^7^ This is in contrast to other reports on primary THR in patients with femoral neck fractures using the postero-lateral approach, the dislocation rate ranged between 13% and 22%^1^,^8^-^10^.

All the cases in our series were assessed according to Harris Hip Score and graded accordingly as Excellent, Good, Fair, Poor and Failure. Our results were similar to the study by Blomfeldt *et al*^7^ who reported mean Harris Hip Score 77.5 in bipolar prosthesis and 82.5 in THR group which was statistically significant with p value 0.011 at four months and 79.4 in the bipolar prosthesis and 87.2 in the THR group, with p value <0.0001.

## Conclusion

We conclude that THR was a better option to treat displaced fracture neck femur in elderly patients based on our shortterm outcome study. We would need to consider the potentially higher morbidity with THR in view of greater blood loss and longer operation time.
